# Mental ill health and factors associated with men’s use of intimate partner violence in Zimbabwe

**DOI:** 10.1186/s12889-018-5272-5

**Published:** 2018-03-20

**Authors:** Mercilene Machisa, Simukai Shamu

**Affiliations:** 1 Gender and Health Research Unit, South African Medical Research Council, Pretoria, South Africa; 2 Foundation for Professional Development, Pretoria, South Africa; 3 School of Public Health, University of Witwatersrand, Johannesburg, South Africa

**Keywords:** Intimate partner violence perpetration, Mental health, Gender attitudes and relationship power, Child abuse, Men

## Abstract

**Background:**

Over the years, researchers have relied on data from women victims to understand the profile on male perpetrators of intimate partner violence (IPV). IPV studies with male participants in the general population are still emerging in Africa. The contribution of mental ill health to IPV perpetration in the general population that has been documented elsewhere is emergent. Notwithstanding, research with male perpetrators is essential to informing effective prevention programmes and interventions. To contribute to the emerging literature on male perpetrators, we conducted a study to estimate the prevalence and factors associated with IPV perpetration by men in heterosexual relationships. We also modelled pathways to IPV perpetration using data from Zimbabwe.

**Methods:**

Data were collected through a nationwide survey employing a random and multi-staged sampling method. We recruited and administered a structured questionnaire to 2838 men aged 18 years and above. IPV was measured using an adapted WHO Domestic Violence Questionnaire. Determinants of IPV measured included child abuse, alcohol abuse, post-traumatic stress disorder (PTSD), depressive symptoms, personal gender attitudes and risky sexual behaviours. Multivariate regression modelling was used to assess factors associated with IPV perpetration. Structural equation modelling was used to explore the underlying pathways to recent IPV perpetration.

**Results:**

Forty one percent of men had perpetrated IPV in their lifetime and 8.8% percent of men perpetrated IPV in the 12 months before the survey. Older, more educated men, men who binge drank, men who were abused as children or experienced other life traumatic experiences were more likely to perpetrate IPV in lifetime. Depressive symptoms and sexual relationship power (were also associated with lifetime IPV perpetration. IPV perpetration in the last 12 months was associated with binge drinking, PTSD and sexual relationship power. The pathways to IPV perpetration in the last 12 months from child abuse to recent IPV were mediated by comorbid PTSD symptoms, depression binge drinking and sexual relationship power.

**Conclusions:**

IPV perpetration was associated with child abuse history, mental ill health, sexual relationship power and personal gender attitudes. Interventions to reduce IPV need to engage men to address gender inequality, mental ill health and reduce alcohol consumption.

## Background

According to the World Health Organisation, one in every three women (35%) across the globe have experienced some form of physical and or sexual violence perpetrated by their male intimate partner in their lifetime [1]. Population-based research on men’s use of violence is emerging and shows higher rates of disclosure of violence compared to research with women’s samples. Studies from Southern Africa have found lifetime prevalence of physical IPV perpetration that are over 30% [2–7]. Factors associated with violence perpetration differ across settings but include socio-economic status, alcohol abuse, and history of child abuse, risky sexual behaviours, educational status, and conservative attitudes condoning violence, relationship conflict and mental ill health. [7–9]. Sociodemographic characteristics associated with IPV in Sub-Saharan Africa are poverty-related and include low education, low income and unemployment [1, 8]. These three factors are related in that higher education helps to enable access to resources including formal or high paying employment. Failure to get education and therefore income render men into feelings of less masculine status and in order to compensate for this, men opt to use violence to assert their power over women or to gain their cooperation [8, 10]. This paper looks at IPV perpetration in an economy that has deprived men of their traditional breadwinning privileges and powers.

Use of violence is more prevalent among men who endorse patriarchal norms supporting male dominance and sexual entitlement is more prevalent among men using violence [11]. Men enacting harmful forms of hyper-masculinity are more likely to perpetrate IPV [6]. There is also evidence showing association between risky sexual behaviours and IPV perpetration [12–15]. Men who perpetrate partner violence were found to be more likely than other men to engage in HIV risk behaviours such as having many sexual partners and practicing transactional sex [15]. It is therefore important to research male perpetration of violence as it is a risk factor for both violence and many other risky sexual behaviours.

Mental ill health, in particular, post-traumatic stress disorder has been shown to increase risk for IPV perpetration [16, 17]. PTSD symptoms are frequently comorbid with depressive symptoms and some studies have established an association between depression and IPV perpetration which can be direct or indirect through binge drinking [12, 18]. Binge drinking or alcohol abuse has also been found to be a risk factor for IPV perpetration in different studies [8, 19–21]. More studies investigating the role of mental ill health in intimate partner violence perpetration have been conducted in developed countries and fewer studies have been conducted in the global south. Data with general population samples and developing countries is emerging [9].

Research conducted in Zimbabwe with women shows that IPV is a prevalent societal problem [22–24]. However, perpetration research with general population samples of men and with a national coverage is lacking. Understanding the profile and characteristics of male perpetrators on a national scale is key in developing programmes and interventions to curb the scourge of violence against women. To fill that gap in literature, we conducted a nationwide household survey to determine the prevalence of lifetime perpetration of sexual or physical IPV and the associated factors among men from the general population in Zimbabwe. This study went beyond analyzing factors associated with IPV perpetration to analyzing the causal pathways to IPV perpetration at two different levels – lifetime IPV and IPV in the last 12 months. This analysis helps to assess further the dynamics and relationships between IPV perpetration and different factors in a man’s life.

## Methods

The study was a cross sectional national household survey conducted in 2012 in Zimbabwe. In the survey, participants were selected through a multi-stage random sampling method, using the 2002 Zimbabwe Population Census as the primary sampling frame. At the first sampling stage districts were sampled from all the 10 provinces using a proportionate to size method. At the district level, the primary sampling unit used were the Enumeration Areas (EAs). An enumeration area, also called a census block or census tract, is the smallest geographic unit for which census information is aggregated, compiled and disseminated. An enumeration area is defined by boundaries described on a sketch map or in a geographic information system (GIS) database [25]. Again, a proportionate to size approach was used to determine the number of EAs selected per district and the national total of 225 EAs. In each selected EA, 20 households were selected and in each household only one eligible man was randomly selected for participation. To be eligible for participation in the study, a man had to be 18 years and above and normally resident in the selected household i.e. sleeping at least four nights a week in the household. Men who were visiting households or who were intoxicated or not in a mental state to complete questionnaires were excluded. While the target sample was 4507 men, only 3274 men in selected households were eligible, available and consented to participate. The survey’s overall response rate was 78%. [26].

### Ethical considerations

The study was conducted in collaboration with and approved by the Government of Zimbabwe’s Ministry of Women Affairs, Gender and Community Development (MWAGCD). Participation in the study was voluntary and prior written informed consent was given by all participants. Interviews were conducted in privacy and participants were assured of confidentiality. To ensure anonymity participants were assigned to random study ID numbers. Unlike studies of women’s experiences of violence where risk for IPV after the interview is high and the need for counselling and support is much more pronounced [27], the planning of this study perceived that there was minimum risk among male participants and their families as a result of this study. However, in case of risk, participants were informed to consult study officials who would assist them. As expected, we did not receive any participant request for help, neither did we identify participants requiring urgent violence or mental health related assistance.

### Data collection

Data was collected through self-administered questionnaires loaded onto a personal digital assistant (PDA). Men aged 18 or older normally resident in the sampled household were recruited and interviewed by trained male researchers using structured questionnaires in a language chosen by the participant from English, Shona or Ndebele.

### The questionnaire

The primary outcome of interest was lifetime sexual or physical IPV perpetration and these were measured using an adapted and pretested version of the WHO Multi-Country Study on Women’s Health and Domestic Violence: Core Questionnaire and WHO Instrument – Version 9 designed for use in developing countries [28]. Table 1 shows the list and flow of questions used in the questionnaire. Sexual IPV was measured using a sub-scale of three items including forcing an intimate partner to have sex or perform sexual acts against their consent by using physical force or other means. Physical IPV was measured using a sub-scale of five items of acts of violence including slapping, throwing dangerous objects, pushing, kicking, hitting, dragging, choking, beating, burning or threatening a current or previous intimate partner with a weapon. Emotional IPV was measured by a series of six acts that described controlling, frightening, intimidating or undermining women’s self-esteem**.** IPV experience in the past 12 months was measured using a follow-up question to each set of sub-scale questions for emotional, sexual and physical IPV as follows: “Have any of these things happened in the past 12 months?” The composite lifetime IPV variable was derived from combining the physical and/or sexual IPV and emotional IPV subscale items and if a man had done any of the acts towards a partner. A similar approach was used for the IPV past 12 months variable.

**Table 1:** Scale and variable items

Variable/ Scale	Average inter-item covariance	Number of items	Scale reliability coefficient	Items
Sexual IPV		3		Have you ever physically forced your current or ex-girlfriend or wife to have sex when she did not want to? Did this happen many times, a few times, once or did it not happen? (Choose one from Never, Once, Few, Many); Have you ever had sex with a current or ex-girlfriend or wife when she did not want to? Did this happen many times, a few times, once or did it not happen? (Choose one from Never, Once, Few, Many); Have you ever forced your current or ex-girlfriend or wife to do something sexual that you found degrading or humiliating? Did this happen many times, a few times, once or did it not happen? (Choose one from Never, Once, Few, Many); Have any of these things happened in the past 12 months? (Yes or No)
Physical IPV		5		Have you ever slapped or thrown something at your current or ex-girlfriend or wife which could hurt her? Did this happen many times, a few times, once or did it not happen? (Choose one from Never, Once, Few, Many); Have you ever pushed or shoved your current or ex-girlfriend or wife? Did this happen many times, a few times, once or did it not happen? (Choose one from Never, Once, Few, Many) Have you ever hit your current or ex-girlfriend or wife? Did this happen many times, a few times, once or did it not happen? (Choose one from Never, Once, Few, Many); Have you ever kicked, dragged, beat, choke or burnt your current or ex-girlfriend or wife? Did this happen many times, a few times, once or did it not happen? (Choose one from Never, Once, Few, Many); Have you ever threatened to use or actually used a gun, knife or other weapon against your current or ex-girlfriend or wife? Did this happen many times, a few times, once or did it not happen? (Choose one from Never, Once, Few, Many); Have any of these things happened in the past 12 months? (Yes or No)
Emotional IPV		6		Have you ever insulted your current or a previous girlfriend or wife or made her feel bad about herself? Did this happen many times, a few times, once or did it not happen? Have you ever belittled or humiliated a current or ex-girlfriend or wife in front of other people? Did this happen many times, a few times, once or did it not happen? (Choose one) Have you ever done things to scare or intimidate a current or ex-girlfriend or wife on purpose for example by the way you looked at her, by yelling and smashing things? Did this happen many times, a few times, once or did it not happen? (Choose one). Have you ever threatened to hurt your current or ex-girlfriend or wife? Did this happen many times, a few times, once or did it not happen? (Choose one) Have you ever boasted about or brought home girlfriends? Did this happen many times, a few times, once or did it not happen? (Choose one). Have any of these things happened in the past 12 months?
Child trauma scale	0.09	13	0.74	I did not have enough to eat; I lived in different households at different times; One or both of my parents were too drunk to take care of me; I spent time outside the home and none of the adults at home knew where I was; I saw or heard my mother being beaten by her husband or boyfriend; I was insulted or humiliated by someone in my family in front of other people; I was told I was lazy or stupid or weak by someone in my family; Someone touched my buttocks or genitals or made me touch them when I did not want to; I had sex with a woman who was more than 5 years older than me; I had sex with someone because I was threatened or frightened or forced;I was beaten at home with a belt or stick or whip or something else which was hard; I was beaten so hard at home that it left a mark or bruise; I was beaten or physically punished at school by a teacher
GEM Scale	0.16	10	0.74	You don’t talk about sex, you just do it; There are times when a woman deserves to be beaten; Changing nappies, giving kids a bath, and feeding the kids are the mother’s responsibility; It is a woman’s responsibility to avoid getting pregnant; A man should have the final word about decisions in his home; Men are always ready to have sex; I would be outraged if I asked him to use a condom; If someone insults me, I will defend my reputation, with force if I have to; To be a man, you need to be tough; Men should be embarrassed if they are unable to get an erection during sex.
Life events score		10	0.71	Traumatic events included imprisonment/detainment, civil unrest/war, serious injury requiring hospitalization, being close to death, witnessing a murder of family or friend, unnatural death of family or friend, witnessing the murder of stranger/s, torture, witnessing or being robbed or car hijacked at gun or knife point and kidnapping.
SRPS	0.13	12	0.77	I become jealous when she wears things that make her look too beautiful; I have more to say than she does about important decisions that affect us; I never tell her who she can see or spend time with; I like to do what I want, even if she doesn’t want me to; When she and I disagree, I get my way most of the time;I like to know where she is most of the time; I expect her to do things for me like my ironing and cooking; I let her know that she is not the only girlfriend I have or could have; When I want sex I expect her to agree; If she asked me to use a condom I would get angry; If she asked me to use a condom I would think she is having sex with other people; If she asked me to use a condom I would hit her.

Sociodemographic characteristics measured included age, level of education attained, employment status in the year before the study. HIV risk characteristics measured included the number of sexual partners in the year preceding the survey and having sex with a sex worker. Determinants of IPV perpetration measured included child abuse, gender attitudes, mental ill health outcomes: PTSD, depression and alcohol abuse, socio-demographics characteristics, relationship characteristics, and HIV risky behaviours. Child abuse was measured through thirteen items of a modified version of the short form of the Childhood Trauma Questionnaire (CTQ) [29] **(**Table 1**)**. Responses to items were Never [1], Sometimes [2], Often [3] and Very often [4]. A continuous child abuse score was created by summing up the response items (Range 13–52). A score greater than 13 was indicative of experience of child abuse. Higher scores indicated more frequent, severe forms of adverse child abuse experiences.

PTSD was measured using 30 items of the Harvard Trauma Questionnaire (HTQ) (Cronbach’s alpha = 0.95) [30]. Responses to items were Not at all (0), A little [1], Quite a bit [2] and Extremely [3]. A continuous PTSD score was created by summing up responses to items (Range 0–90). Higher than 30 scores were indicative of presence of PTSD symptoms.

Depressive symptoms were measured using the 20-item Centre for Epidemiologic Studies Depression (CES-D) self-report measure (Cronbach’s alpha =0.81) [28]. Responses to items were “Rarely or none of the time (0), Some or a little of the time (1–2 days) [1], Moderate amount of time (3–4 days) [2] and Most or all of the time (5–7 days) [3]”. A continuous CES-D score was obtained by summing up responses to items (Range 0–60). A score higher than 16 was indicative of more severe depressive symptoms.

Binge drinking was measured using the Alcohol Use Disorders Identification Test (AUDIT) scale [31]. We used the question “How often do you have 5 or more drinks on one occasion?” Responses were “never, less than monthly, monthly, weekly and daily or almost daily.” A man was said to be a binge drinker if he consumed 5 or more drinks on one occasion on a weekly, daily or almost daily basis.

Experience of traumatic life events was measured through an adapted Life Events Checklist from the PTSD Checklist **(**Table 1**)**) [30]. Responses to items were either Yes [1] or No (0). A continuous life events score was created by summing up the response items (Range 0–10). The traumatic live events score increased with the number of traumatic events exposures.

We used the Gender Equitable Men Scale (GEMS) to measure attitudes toward gender norms in intimate relationships and differing social expectations for men and women (Table 1) [32]. Responses were “Strongly agree [1], Agree [2], Disagree [3] and Strongly Disagree [4]” (Range 10–40). Higher scores were indicative of progressive and more equitable gender attitudes.

We used an adaptation of the Sexual Relationship Power Scale (SRPS) to measure power within sexual relationships. The scale consisted of relationship control items that predict sexual and physical violence, condom use and partner infidelity (Table 1) [33]. Responses were “Strongly agree [1], Agree [2], Disagree [3] and Strongly disagree [4]” (Range 12–48). Higher scores were indicative of more equitable sexual relationship power.

### Data analysis

For this secondary analyses we excluded men who had never been in a heterosexual intimate relationship (*n* = 436). All data analyses were done using Stata version 13 considering the survey’s multi-stage sample design. We used cross tabulations to describe the prevalence of lifetime and past 12 months IPV perpetration against the different sociodemographic variables, binge drinking, and sexual partners **(**Table 2**)**. For the continuous variables - Child abuse, CESD, PTSD, Life events, GEM and SRPS Scores, we used t-tests to compare the means across perpetrator vs non-perpetrator groups for both the lifetime IPV and past 12 months IPV **(**Table 3**)**.

**Table 2:** IPV perpetration disaggregated by categorical variables

	Total		Never IPV in lifetime		IPV once or more in lifetime		*P* Value	No IPV in past 12 months		IPV in past 12 months		*P* value
	*n*	%	*n*	%	*n*	%		*n*	%	*n*	%	
Age: 18–29 years	1038	36.6	685	40.9	353	30.4	0.0005	938	36.3	100	40	0.147
30–44 years	1090	38.4	613	36.6	477	41.1		985	38.1	105	42	
45+ years	709	25	377	22.5	332	28.6		664	25.7	45	18	
Education: Secondary school incomplete	1011	35.7	559	33.4	452	39	0.1721	927	35.9	84	33.7	0.7713
Secondary school complete only	1473	52	904	54	569	49.1		1337	51.7	136	54.6	
Secondary school complete and higher	349	12.3	210	12.6	139	12		320	12.4	29	11.7	
Employed in the last 12 months	1201	42.5	688	41.2	513	44.3	0.2319	1065	41.3	136	54.4	0.0007
Binge drinking	610	22.1	269	16.4	341	30.2	0.0002	522	20.7	88	35.9	< 0.0001
Sexual partners in past 12 months: None	213	8.1	130	8.6	83	7.5	0.0618	199	8.4	14	5.7	0.0119
One	1649	63	965	64.2	684	61.5		1513	63.8	136	55.5	
Two or more	754	28.8	409	27.2	345	31		659	27.8	95	38.8

**Table 3:** IPV disaggregated by continuous variables

	Never IPV in lifetime				IPV once or more in lifetime				*P* Value
	*n*	Mean	95% CI		*n*	Mean	95% CI		
Child trauma score	1543	17.9	17.7	18.1	1074	18.9	18.7	19.16	< 0.0001
PTSD score	1564	7.4	6.8	7.9	1094	11.2	10.4	12.06	< 0.0001
CESD Score	1593	9.3	8.9	9.7	1102	11.6	11.1	12.08	< 0.0001
Life events score	1619	1.2	1.2	1.3	1110	1.7	1.6	1.85	< 0.0001
GEM Score	1568	24.8	24.5	25.0	1118	24.0	23.7	24.26	< 0.0001
SRPS	628	31.1	30.7	31.5	1072	29.5	29.2	29.80	< 0.0001
	No IPV in past 12 months				IPV in past 12 months				*P* value
	*n*	Mean	95% CI		*n*	Mean	95% CI		
Child trauma score	2387	18.2	18.0	18.4	230	19.3	18.8	19.8	0.0001
PTSD Score	2415	8.5	8.0	9.0	243	13.2	11.3	15.1	< 0.0001
CESD Score	2453	10.0	9.7	10.3	242	13.0	11.9	14.1	< 0.0001
Life events score	2486	1.4	1.3	1.5	243	1.7	1.4	1.9	0.0395
GEM Score	2440	24.5	24.4	24.7	246	23.5	22.9	24.0	0.0004
SRPS	1464	30.4	30.1	30.6	236	28.5	27.9	29.2	< 0.0001

To test for factors associated with lifetime and past 12 months IPV, we built logistic regression models. We included characteristics that had a *p*-value less than 0.2 in bivariate analyses into regression models. We controlled for the effect of sociodemographic variables (age, education and working for income) in models. We then used a stepwise backward elimination approach to eliminate non-significant associations until final models were arrived at that were parsimonious **(**Table 4**)**.

**Table 4:** Factors associated with lifetime and past 12 months IPV perpetration

	a. Logistic regression model of factors associated with lifetime IPV perpetration				b. Logistic regression model of factors associated with past 12 months IPV perpetration			
	aOR	95% CI		P value	aOR	95% CI		*P* value
Age group: 18–29 years	1.0				1.0			
30–44 years	1.57	1.18	2.10	0.005	0.73	0.47	1.14	0.145
45+ years	2.00	1.26	3.18	0.008	0.61	0.27	1.39	0.208
Education: Secondary school incomplete	1.0				1.0			
Secondary school complete only	1.22	0.80	1.89	0.309	1.23	0.84	1.81	0.252
Secondary school complete and higher	1.87	1.20	2.91	0.011	1.18	0.50	2.77	0.669
Child abuse score	1.12	1.05	1.21	0.003	1.05	0.99	1.12	0.114
Binge drinking	1.90	1.44	2.48	< 0.0001	1.81	1.32	2.48	0.002
CESD score	1.05	1.01	1.08	0.01	1.03	0.98	1.08	0.219
PTSD score	1.02	0.99	1.06	0.133	1.02	1.00	1.03	0.026
Life events score	1.18	1.05	1.34	0.011	1.02	0.93	1.13	0.626
SRPS	0.94	0.89	1.00	0.046	0.94	0.88	0.99	0.032
GEM score	1.04	0.99	1.10	0.122	1.00	0.96	1.06	0.771

Further to the logistic regression modelling, we employed Structural Equation Modelling (SEM) with maximum likelihood estimation to investigate the underlying pathways of factors associated with recent/or 12 months IPV perpetration. Our a priori model was that there were significant inter-relationships between variables. Child abuse could be associated with life traumatic events, mental ill health, gender scales and IPV perpetration. Gender scales could predict IPV perpetration. Mental ill health symptoms could be comorbid and predict IPV perpetration. We fitted an SEM model based on known assumptions from the literature (for example that gender imbalance could predict IPV perpetration [34]) and the results from the regression model i.e binge drinking, PTSD and sexual relationship power predicted past 12 months IPV perpetration. We specified model paths and allowed the errors of indicator variables to co-vary when the covariance improved the model fit and was theoretically justifiable. We covaried SRPS with GEM scores, binge drinking with GEM score and age with education. We estimated the model and tested for goodness of fit of the model by assessing the Comparative Fit Index and the Root mean squared error of approximation (RMSEA). The final model’s standardized parameter estimates are displayed in Table 5.

**Table 5:** SEM model statistics

	Standardised co-efficient	SE	z	95% CI		*P* value
Age→ Child abuse	0.06	0.02	3.20	0.02	0.09	0.001
Child abuse → Gem Score	−0.17	0.02	−9.37	−0.21	−0.14	< 0.0001
Education → Gem Score	0.14	0.02	7.69	0.10	0.17	< 0.0001
Child abuse → PTSD	0.32	0.02	18.29	0.29	0.36	< 0.0001
Age →PTSD	0.07	0.02	3.95	0.03	0.10	< 0.0001
Child abuse →CESD	0.07	0.02	4.25	0.04	0.11	< 0.0001
PTSD →CESD	0.53	0.01	37.27	0.50	0.56	< 0.0001
Child abuse →SRPS	−0.16	0.02	−6.28	−0.20	− 0.11	< 0.0001
Education→ SRPS	0.15	0.02	6.71	0.11	0.19	< 0.0001
CESD →Binge drinking	−0.04	0.02	−2.10	−0.07	0.00	0.036
Age → Binge drinking	0.07	0.02	3.90	0.04	0.11	< 0.0001
Education → Binge drinking	0.06	0.02	2.99	0.02	0.09	0.003
PTSD → IPV past 12 months	0.10	0.02	4.92	0.06	0.13	< 0.0001
CESD → IPV past 12 months	0.07	0.02	3.26	0.03	0.12	0.001
SRPS→ IPV past 12 months	−0.09	0.02	−4.43	−0.13	−0.05	< 0.0001
Binge drinking→ IPV past 12 month	0.10	0.02	5.29	0.06	0.13	< 0.0001
Deviation statistics	Standardised co-efficient	SE	95% CI			
Child abuse	0.997	0.002	0.992	1.001		
GEM Score	0.951	0.008	0.936	0.965		
PTSD score	0.890	0.012	0.867	0.913		
CESD score	0.690	0.014	0.662	0.718		
SRPS score	0.952	0.010	0.934	0.971		
Binge drinking	0.993	0.003	0.987	0.999		
IPV past 12 months	0.973	0.006	0.962	0.985		
Model fit statistics						
p > chi^2^	< 0.0001					
RMSEA	0.049					
CFI	0.939					
TLI	0.881					

## Results

The sample comprised 2838 men who had been in heterosexual intimate relationships: 41% perpetrated IPV in lifetime and 8.8% perpetrated IPV in the 12 months before the survey. One in ten men had a score of 30 or above on the PTSD scale, 20.5% had a CESD score of 16 or more, 88.7% had experienced some form of child abuse.

Table 2 shows sociodemographic variables by IPV perpetration. More men in the ages 30–44 years reported perpetrating lifetime violence than their younger or older counterparts (*p* = 0.0005). There was however no significant difference in IPV perpetration in past 12 months by age (*p* = 0.147). There was no significant difference in perpetration of lifetime or past 12 months IPV by men’s highest educational attainment. More men who were employed in the last 12 months perpetrated violence than men who were not employed in the same period (*p* = 0.0007). A higher proportion of men who worked to earn income in the past 12 months perpetrated IPV in a similar period**.** A higher proportion of men who binge drank perpetrated IPV in lifetime (*p* = 0.0002) and in the past 12 months (*p* < 0.0001) than those who did not binge drink. A higher proportion of men who had 2 or more partners in the past 12 months perpetrated IPV in the same period than those who had less than two sexual partners (*p* = 0.0119)**.**

Table 3 shows mean life experiences and gender norms/equity variables in continuous form and disaggregated by IPV perpetration. The mean scores on the child abuse scale, CESD, PTSD and Life Events Checklist for men perpetrating IPV in lifetime and past 12 months were significantly higher than those of men who never perpetrated IPV. This shows higher prevalence of child abuse history and mental ill health symptoms among perpetrators compared to non-perpetrators. The mean scores on the GEM and SRPS scales were significantly lower for men who perpetrated IPV in lifetime and in past 12 months compared to men who did not. This showed more gender equitable attitudes among non-perpetrators.

Table 4 shows factors associated with lifetime and past 12 months IPV perpetration in adjusted Odds Ratios (aOR). Lifetime IPV perpetration was associated with age, education status, child abuse, binge drinking, depressive symptoms, traumatic life events and sexual relationship power **(**Table 4a**)**. Older men were more likely to perpetrate lifetime IPV compared to the 18–29 year olds. The risk for lifetime IPV perpetration increased with child trauma, traumatic life events, depression and PTSD symptom scores. Men who binge drank were 89% more likely to perpetrate IPV in lifetime compared to men who did not binge drink. Higher scores on the SRPS scales were protective of lifetime IPV perpetration.

Factors associated with IPV in past 12 months were binge drinking, PTSD and sexual relationship power **(**Table 4b**)**. Men who binge drank were 81% more likely to perpetrate IPV compared to the men who did not. Risk for IPV perpetration in past 12 months increased with PTSD symptoms. Higher scores on the SRPS scale were protective of past 12 months IPV perpetration **(**Table 4b**)**.

**Fig. 1:**
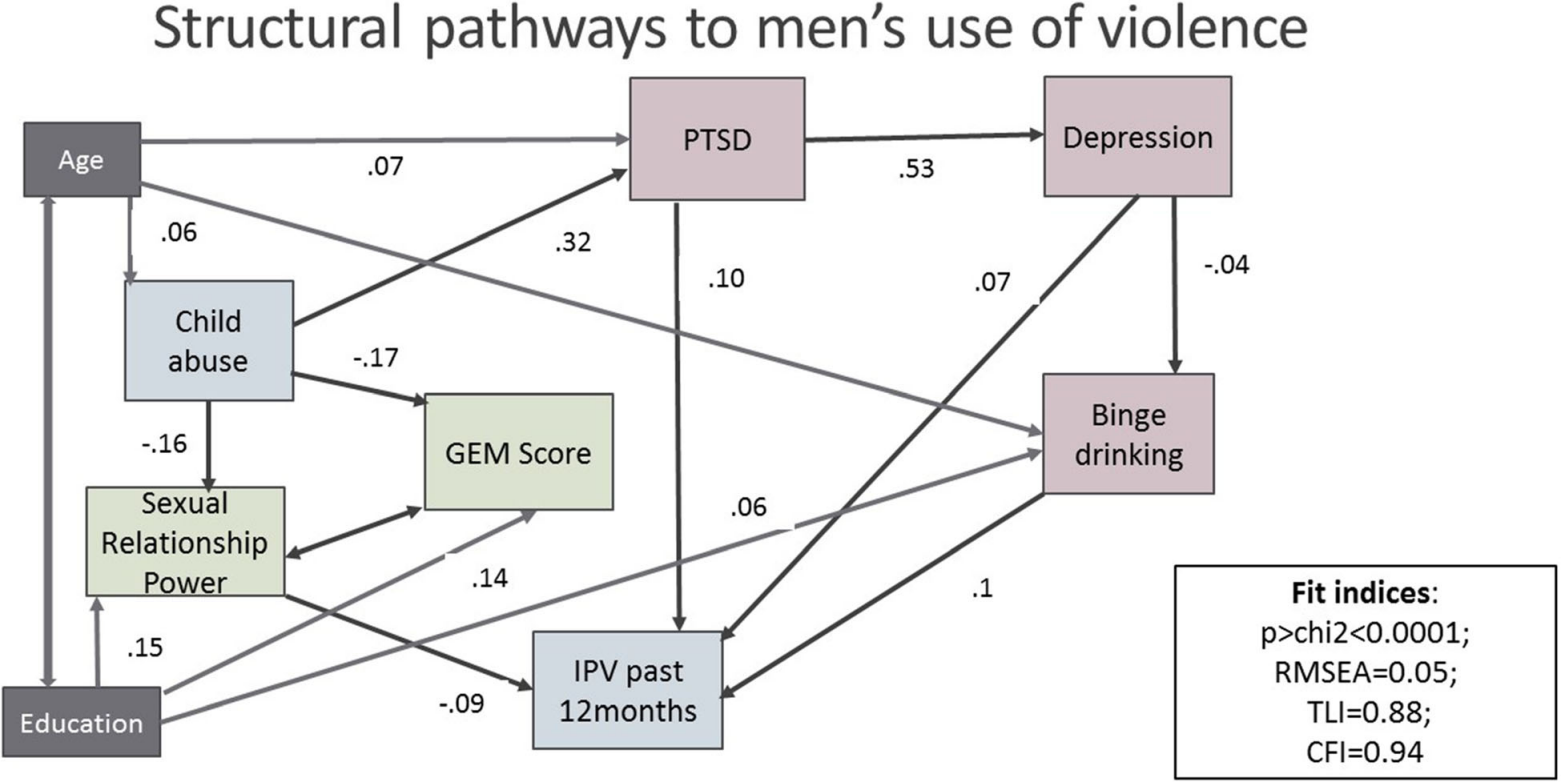
Structural pathways to men’s use of violence

Figure 1 and Table 5 shows the results from the SEM. Child abuse has direct effects on gender attitudes, sexual relationship power and depressive symptoms. Educational status had direct effects on gender attitudes. Age had direct effects on binge drinking and PTSD. Depression was comorbid with PTSD and binge drinking: PTSD had direct effects on depression, depression had effects on binge drinking. PTSD, binge drinking and sexual relationship power had effects on past 12 months IPV. Binge drinking co-varied with gender attitudes. Gender attitudes co-varied with sexual relationship power. The final model had acceptable fit. (CFI = 0.94; TLI =0.88; RMSEA = 0.05); *p* value = < 0.0001).

## Discussion

The study aimed to investigate the factors associated with men’s use of violence against intimate partners. Factors associated with lifetime perpetration included child abuse, high gender inequity attitudes, binge drinking, life traumatic events, depressive symptoms and sexual relationship power. Factors associated with recent IPV perpetration included binge drinking, PTSD symptoms and less sexual relationship power. Path modelling of variables showed that child abuse had direct effects on PTSD, depression, gender attitudes and sexual relationship power. Sexual relationship power, binge drinking, PTSD and CESD had direct effects on IPV in past 12 months.

The findings show the contribution of child abuse to IPV perpetration in a context where corporal punishment at home and in school institutions is legalized through Article 241 of the Criminal Law (Codification and Reform) Act 2004 and the Article 7 of the Children’s Act 1972 [35]. Child abuse was also associated with mental ill  health and had effects on PTSD, depressive symptoms and binge drinking, consistent with global literature on the long-term effects of child physical, sexual and emotional abuse [36–39].

Child abuse also had effects on gender attitudes and sexual relationship power. This is consistent with literature showing that men who were  abused in childhood are more likely to have witnessed parental violence and have been socialized into gender inequitable norms leading to their  use violence in their intimate  relationships [40]. These findings show that child abuse is a key determinant of negative outcomes in adult life hence is a key area of action for preventing mental ill  health, negative attitudes development and IPV perpetration. More importantly these findings show evidence of the need to change societal discourse on child beating and corporal punishment because of its negative contribution to intergenerational transmission of violence. Abolishing all forms of corporal punishment may be a good starting point towards a violence free environment. The findings also show evidence for the need to protect children from all forms of abuse including sexual abuse that have protracted effects in the life course.

Mental ill health among men was also shown to be a determinant of violence perpetration. Consistent with other research showing PTSD as a risk factor for IPV  perpetration, [9, 18, 41] we found an association between PTSD and recent IPV perpetration. There was also relatively high prevalence of depression among men in this study that was associated with lifetime perpetration of IPV but not recent IPV. The societal context of depression could have been driven by more factors than were collected in this study for example the poverty and economic recession that the country faced for over a decade. There is evidence that poverty, job losses and other economic stressors are key determinants of depression [42].

Notwithstanding, the data showed comorbidities between binge drinking and depression and that binge drinking fully-mediated the relationship with violence perpetration. Further investigations are necessary to understand these relationships because they provide an opportunity for intervening to reduce violence. The association of binge drinking with IPV perpetration can be explained by the distortion of perceptions of cues and lowering of inhibitions which lead to aggression in a relationship [43]. Considering this relationship there is potential for alcohol reduction programmes in reducing violence in intimate relationships. Health promotion geared to encourage men to seek treatment for mental ill health and counselling services are essential for violence prevention. Because the country’s health sector is struggling there will be need for the  prioritization of community based social support mechanisms to help men deal with their mental health challenges.

The data also shows that inequitable gender norms and behaviours associated with IPV are a key driver of violence perpetration. Gender norms are the result of socialization of individuals based on social expectations for appropriate behaviours of males and females. Society defines context specific models of manhood and these are passed onto boys and men through cultural processes. When norms are inequitable they negatively affect relationships with women and are often associated with partner violence [32]. Sexuality education coupled with gender transformative programmes for men and boys are critical to reduce partner violence. Existing community based gender equitable norms creation and reinforcement programmes in Zimbabwe need to be strengthened [44, 45].

Inequitable gender attitudes were also associated with sexual relationship power. The SRPS was developed to measure women’s ability to negotiate safer sex practices and HIV risk as it relates to gender-based imbalances in the relationship [33]. The association of the GEM Score with sexual relationship power shows that inequitable gender norms are important factors in sexual health decision making and HIV risky behaviours [32]. In this regard work in transforming gender attitudes of men, in addition to violence reduction, may result in healthy relationships which are empowering to female partners in terms of sexual decision-making, and reproductive health choices [10, 46]. There is also need to consider working with men as agents of social change or as champions for nurturing gender equitable attitudes through modelling gender equitable relationships for youths and other men to adopt [45].

The relationship between loss of economic power and perpetration of violence has been discussed in many settings [47]. This explanation enables us to understand our findings in the context of the Zimbabwean prolonged economic recession characterized by high unemployment and more informal than formal jobs. Such an economy has subsequently changed concepts of gender roles leading to women becoming more vulnerable to violence as men use violence instead to exert their power over women. Women have become key players in the informal sector and changed significantly their contribution to family welfare. This has led to changes in gender dynamics that has left men feeling less powerful and their manhood threatened. The loss of power by men may lead to men resorting to violence to exert this control. Qualitative work is necessary to further investigate the influences of the economy on violence in relationships.

There are several limitations to the study. The cross-sectional design limits the study in establishing temporality but the path analysis assists us to know how the factors are ordered and the directions in which variables are linked. We cannot conclude on the relationship between depression and IPV as there may be other factors associated with depression that were not measured in this study, for example, economic stressors. The observed relationships in this study are those that can be concluded based on the included variables only. The paper is based on men’s self-report data which may have been affected by response bias such as unwillingness to disclose socially undesirable behaviors or characteristics. Nevertheless, the study methodology has its strengths which include having a nationwide coverage and a community based sample with sufficient power to generalize the findings.

## Conclusions

The study found high levels of lifetime male perpetration of intimate partner violence. Lifetime perpetration of IPV was associated with child abuse, high gender equity attitudes, binge drinking, life traumatic events, depressive symptoms and sexual relationship power. Factors associated with recent IPV perpetration were binge drinking, PTSD symptoms and less sexual relationship power. The causal pathway analysis found experiences of child abuse having a direct effect on PTSD, depressive symptoms, gender attitudes and sexual relationship power. PTSD, depressive symptoms, binge drinking and sexual relationship power had a direct effect on IPV in past 12 months.

The public health implications of these findings are that primary prevention of IPV perpetration must focus on reducing child abuse by addressing negative parenting practices. Secondary prevention needs to address changing gender norms, promoting responsible behaviours, gender equitable masculinities as well as addressing mental ill health symptoms and alcohol abuse.

## Abbreviations

aOR: Adjusted Odds Ratio
AUDIT: Alcohol Use Disorders Identification Test
CES-D: Centre for Epidemiologic Studies Depression
CFI: Confirmatory Fit Index
CI: Confidence level
CTQ: Childhood Trauma Questionnaire
EA: Enumeration area
GEMS: Gender Equitable Men Scale
HIV: Human immunodeficiency virus
HTQ: Harvard Trauma Questionnaire
ID: Identity number
IPV: Intimate partner violence
MWAGCD: Ministry of Women Affairs, Gender and Community Development
PDA: Personal digital assistant
PTSD: Post-traumatic stress disorder
RMSEA: Root mean squared error of approximation
SEM: Structural Equation Model
SRPS: Sexual Relationship Power Scale
TLI: Tucker-Lewis Index
WHO: World Health Organization
